# Proteomic characterization of chromosomal common fragile site (CFS)-associated proteins uncovers ATRX as a regulator of CFS stability

**DOI:** 10.1093/nar/gkz510

**Published:** 2019-06-10

**Authors:** David Pladevall-Morera, Stephanie Munk, Andreas Ingham, Lorenza Garribba, Eliene Albers, Ying Liu, Jesper V Olsen, Andres J Lopez-Contreras

**Affiliations:** 1 Department of Cellular and Molecular Medicine, Center for Chromosome Stability and Center for Healthy Aging, University of Copenhagen, Copenhagen 2200, Denmark; 2 Proteomics Program, Novo Nordisk Foundation Center for Protein Research, Faculty of Health and Medical Sciences, University of Copenhagen, 2200 Copenhagen, Denmark

## Abstract

Common fragile sites (CFSs) are conserved genomic regions prone to break under conditions of replication stress (RS). Thus, CFSs are hotspots for rearrangements in cancer and contribute to its chromosomal instability. Here, we have performed a global analysis of proteins that recruit to CFSs upon mild RS to identify novel players in CFS stability. To this end, we performed Chromatin Immunoprecipitation (ChIP) of FANCD2, a protein that localizes specifically to CFSs in G2/M, coupled to mass spectrometry to acquire a CFS interactome. Our strategy was validated by the enrichment of many known regulators of CFS maintenance, including Fanconi Anemia, DNA repair and replication proteins. Among the proteins identified with unknown functions at CFSs was the chromatin remodeler ATRX. Here we demonstrate that ATRX forms foci at a fraction of CFSs upon RS, and that ATRX depletion increases the occurrence of chromosomal breaks, a phenotype further exacerbated under mild RS conditions. Accordingly, ATRX depletion increases the number of 53BP1 bodies and micronuclei, overall indicating that ATRX is required for CFS stability. Overall, our study provides the first proteomic characterization of CFSs as a valuable resource for the identification of novel regulators of CFS stability.

## INTRODUCTION

Common fragile sites (CFSs) are chromosomal regions highly susceptible to break, and thus pose a serious threat to genomic integrity (1). These genomic regions are present in all individuals and can give rise to chromosomal abnormalities, including small deletions and insertions, and translocations (2,3). This genomic instability that arises at CFSs is a hallmark of cancer, and can indeed be observed in early stages of tumorigenesis (4,5). Several properties of CFSs contribute to the fragile nature of these genomic regions. First, many CFSs harbor long genes, such as *FHIT* (in FRA3B) and *WWOX* (in FRA16D), increasing the probability of collision between the replication and transcription machineries, which may ultimately result in replication fork collapse (6–10). Second, some CFSs contain few replication origins, requiring replication forks to cover longer regions and rendering fewer opportunities to rescue replication upon replication stress (RS) or fork stalling (11). Third, some CFSs encompass repetitive sequences and regions that are particularly A-T rich, which can form secondary DNA structures, further complicating DNA replication and thus contributing to fragility (12). Moreover, it is well-known that CFSs complete replication very late in S-phase (13), limiting the time available to repair challenged CFSs prior to mitosis, thereby further increasing their fragility. Importantly, these inherent complications of replicating CFSs are exacerbated in conditions of mild RS which occurs upon oncogenic transformation or experimentally with low doses of the polymerase inhibitor aphidicolin (APH), that slows down replication (13–15).

The factors that have been implicated in CFS maintenance and repair are largely known from other DNA repair pathways, including double strand break (DSB) repair (i.e. ATM, MRN complex and BRCA1) (16,17) the RS response (CHK1 and ATR) (18,19) and interstrand crosslink repair, such as the Fanconi Anemia proteins (20). Despite these defense mechanisms, some perturbed CFSs are not resolved in a timely manner. Consequently, upon entry into mitosis, a next line of defense is set in motion, involving cleavage of the CFSs by MUS81-EME1 endonuclease to permit mitotic DNA synthesis (MiDAS) and thereby completion of CFS replication (21). Occasionally, all lines of defense fail, resulting in chromosomal aberrations that are transmitted to daughter cells, and can give rise to pathological conditions such as cancer. Given the hazards that these fragile regions can pose, and their ubiquitous nature in human cells, the study of novel factors regulating CFS stability may increase the understanding of the diseases in which they are involved.

Typically, studies of CFS maintenance proteins have been restricted to hypothesis driven approaches using immunofluorescence-based (IF) techniques, which are limited by the availability of specific antibodies. In this study, we aimed to characterize the proteins that localize to CFSs using an unbiased approach, in order to identify novel players that regulate CFS stability. To this end, we took advantage of the latest Orbitrap Mass Spectrometry (MS) (22) developments in combination with chromatin immunoprecipitation (ChIP) of FANCD2, to map the proteins that are recruited to CFSs when challenged. FANCD2 is a DNA repair protein from the Fanconi Anemia pathway that localizes to challenged CFSs in G2 and mitosis (23,24), and is essential to ensure their efficient replication (25). By combining the FANCD2 ChIP with stable isotope labeling of amino acids in cell culture (SILAC) (26) for accurate quantification, we generated the first map of the CFS proteome. Using this quantitative interaction proteomics approach, we identified many proteins that are already known to play a role in CFS maintenance, including factors involved in chromosome organization, DNA replication and DNA repair. Furthermore, we mapped proteins which, to date, are uncharacterized in the context of CFS regulation. Of the prominent and unknown CFS associated proteins, we characterized the role of the chromatin remodeler ATRX. Our study demonstrates that ATRX localizes to a subset of CFSs, and is relevant to prevent CFS instability. Overall, we believe that our approach and the resource that we provide in this study will serve as a platform for future investigations to gain insights into the biology of these poorly characterized genomic regions.

## MATERIALS AND METHODS

### Cell culture

HeLa cells were grown in Dulbecco's modified Eagle's medium (DMEM) (GIBCO) supplemented with 10% fetal bovine serum (FBS) (Life Technologies) and 1% of penicillin/streptomycin (Life Technologies). For the proteomics experiments, HeLa S3 were used as HeLa is telomerase positive, and these suspension cells facilitated culturing of the large number of cells required for ChIP-MS. For SILAC labeling (26), HeLa S3 were grown in SILAC RPMI (Roswell Park Memorial Institute) 1640 media (Biowest) supplemented with 10% dialyzed FBS (Sigma), 100 μg/ml penicillin (Invitrogen), 100 μg/ml streptomycin (Invitrogen), 2 mM l-glutamine (Gibco) and one of the following three labeling combinations: (i) natural *light* variants of the amino acids (Lys0, Arg0) (Sigma); (ii) *medium* variants of amino acids {L-[2H4]Lys (+4) and L-[13C6]Arg (+6)} (Lys4, Arg6) and (iii) *heavy* variants of the amino acids {L-[13C6,15N2]Lys (+8) and L-[13C6,15N4]Arg (+10)} (Lys8, Arg10). Medium and heavy variants of amino acids were purchased from Cambridge Isotope Laboratories. This medium will henceforth be referred to SILAC RPMI. All cell lines were maintained at 37°C in a 5% CO_2_ incubator.

### Cell synchronization

For MS experiments, asynchronous medium and heavy labeled HeLa S3 cells were arrested in G2/M by 16 h treatment with 4 mM thymidine (Sigma, T9250), followed by 7.5 h release into fresh SILAC RPMI, which is thereafter supplemented with 100 μg/μl nocodazole (Sigma, M1404), with or without APH (0.2 μM) (Sigma, A0781) or DMSO (Sigma, D2438) for 20 h. For pilot experiments thymidine was used at 2 mM, nocodazole treatment was for 16 h, and APH at 0.4 μM.

### Flow cytometry analysis

HeLa S3 cells were harvested by centrifugation (400 × *g* for 4 min) and washed once with 1× PBS. Subsequently cells were fixed with 70% ice-cold ethanol and incubated for at least 30 min at 4°C. Fixed cells were washed two times with 1% BSA in PBS and incubated with 100 μg/ml ribonuclease A (Sigma, R6513) and propidium iodide (20 μg/ml) (Sigma, P4864) at 37°C for 1 h. DNA content was determined by flow cytometry with Fortessa 3 lasers (BD Biosciences) and the analysis was performed with the Flowjo software.

### ChIP for MS analysis

SILAC-labeled HeLa S3 cells were grown and synchronized accordingly to the synchronization protocol described above. After synchronization ∼70 × 10^6^ cells were used for each ChIP experiment. Cells were harvested by centrifugation for 3 min (900 × *g*) and washed three times with ice cold PBS. Cells were permeabilized in ice cold 0.2% Triton X-100 (Sigma, T8787) in PBS supplemented with protease inhibitor (complete inhibitor cocktail tablet) (Roche) for 5 min on ice. Subsequently, cells were pelleted, washed twice with ice cold PBS and cross-linked in 4% formaldehyde (VWR Chemical) for 15 min at room temperature (RT). Cross-linked cells were washed twice with ice cold PBS and resuspended in lysis buffer (150 mM NaCl, 1% NP-40, 0.5% sodium deoxycholate, 0.1% SDS, 25 mM Tris–HCl and 5 mM EDTA) supplemented with 5 mM β-glycerolphosphate, 5 mM NaF, 1 mM sodium orthovanadate and complete inhibitor cocktail tablet (Roche). Samples were sonicated using a Bioruptor^®^ Pico (Diagenode B01060010) for chromatin fragmentation. Briefly, samples were collected in microcentrifuge tubes containing 50 mg of beads (provided by Diagenode for sonication) and sonicated at 4°C for 40 cycles with 30 s on and 30 s off. 4 μg of FANCD2 (NB100–182SS) or control rabbit IgG (Sigma-Aldrich) antibody was added to cell lysates and the mixture was incubated for 2 h with rotation at 4°C. Chromatin bound to the antibody was then immunoprecipitated using 60 μl of protein G agarose beads slurry (Pierce™ 20398) which had been washed three times in lysis buffer. Samples were immunoprecipitated overnight with rotation at 4°C. The beads were pelleted and washed once with lysis buffer, and the three samples corresponding to the three different SILAC conditions were pooled. Subsequently, beads were washed three times with lysis buffer, crosslinking was reversed and proteins were eluted with 4% SDS, 25 mM Tris supplemented to 100 mM dTT for 1 h at 95°C.

### In gel digestion

The eluted proteins were separated by SDS-page on NuPage 4–12% Bis–Tris protein Gel (Novex, Thermo Fisher Scientific). The proteins were visualized by coomassie using the Colloidal Blue Staining Kit (Invitrogen). Gel bands were cut, destained (50% ethanol in 25 mM ammonium bicarbonate (ABC)) and dehydrated in 96% ethanol. Subsequently, the samples were incubated with 10 mM DTT (in 25 mM ABC) for 30 min, followed by 30 min incubation with 55 mM CAA (in 25 mM ABC). After two washes in 25 mM ABC, and dehydration (in 96% ethanol) proteolysis was performed with trypsin (Sigma) (1.5 μg/ml) overnight. Trypsin activity was quenched with trifluoroacetic acid (TFA) at a final concentration of 1%. The eluate was collected, and the gel pieces were sequentially incubated with Washing solution A (30% (v/v) acetonitrile (ACN), 3% (v/v) TFA in milliQ), Washing solution B (80% (v/v) ACN, 0.5% (v/v) acetic acid in milliQ) and finally with 100% ACN. Samples were collected after incubation with each buffer for 30 min. The ACN was removed by vacuum centrifugation and the resulting peptides were loaded on C-18 STAGE-tips (27). Stagetipping was performed as (28).

### Nanoflow LC–MS/MS

For LC–MS/MS analysis, peptides were eluted from STAGE-tips with 40% ACN in 0.5% formic acid, ACN was removed by vacuum centrifugation and the peptides were reconstituted in 5% ACN, 1%TFA. Peptides were analyzed with an Easy-nLC 1000 system coupled to the Q Exactive HF-X instrument (Thermo Fisher Scientific) through a nanoelectrospray ion source. Peptide separation was performed on an analytical column (inner diameter 75 μm), with 1.9 μm C18 beads (Dr Maisch, packed in-house), at 40°C with an integrated column oven (PRSO-V1, Sonation GmbH). The peptides were eluted on a 15 cm column over 30 min at a flow rate of 350 nl/min from 10% to 80% buffer B (80% ACN, 0.5% formic acid). MS data was acquired in data-dependent mode (DDA), with positive polarity and 30 s dynamic exclusion window. Full scans were acquired with 60 000 resolution, at 350–1400 *m*/*z* scan range, with an ion target value of 3e6 and 45 s maximum injection time. MS/MS were acquired with a top 6 method, scans were acquired at 30 000 resolution, with a ion target of 1e5 and scan range of 200–2000 *m*/*z*. All fragmentation was performed in parallel acquisition with higher-collision dissociation (HCD). Samples from the pilot experiment were analyzed on a Q-Exactive HF, using a top 10 method, with fulls scans acquired at 120 000 resolution, maximum injection time of 20 ms, at a scan range of 300–1750 *m*/*z*. MS/MS scans were acquired at 60 000, ion target value of 2e5, maximum injection time of 110 ms.

### Database search and quantification

All raw LC–MS/MS files were analyzed in the MaxQuant software suite v. 1.6.0.1 (29), with the integrated database search engine Andromeda (30), against the Uniprot database. Common contaminants were included in the database and excluded upon data analysis. Searches were performed with the match between runs options. Cystein carbidomethylation was set as a fixed modification, while methionine oxidation and N-terminal acetylation were set as variable modifications. False discovery rate was set to 1% for peptide spectral matches (PSMs) and protein level, calculated using the decoy database strategy.

### Bioinformatics analysis of MS data

We required that all proteins groups were identified with at least two peptides for inclusion in further analysis. GO term enrichment analysis was performed using DAVID database (31,32) and the enrichment was calculated against the human genome as reference. Functional network analysis was mapped using the STRING database in Cytoscape (33), requiring a minimum confidence score of 0.4.

### RNA interference

HeLa cells were reverse-transfected in DMEM supplemented with 10% FBS with the indicated siRNAs using DharmaFECT 1 transfection reagent (Dharmacon, T-2001-01) and following the manufacturer's instructions. The media was changed the day after transfection. The final siRNA concentration used was 20 nM and experiments were performed 72 h after transfection. The sequences of the sense strand of siRNA duplexes are UAACGACGCGACGACGUAA (siCtrl#1 control 1); CGUACGCGGAAUACUUCGA (siCtrl#2, control 2); CAAUGGUAUUGCUACAUUU (siATRX#1); GUGGGCUGAAGAAUUUAAU (siATRX#2); GCACCGUAUUCAAGUACAA (siFANCD2#1); GAUAAGUUGUCGUCUAUUA (siFANCD2#2); CGUGCCCACUCUCUGUUUU (siDAXX#1); CAGAAACAUUAAUAAACAAUA (siDAXX#2).

### Western blotting

To obtain protein extracts for SDS-PAGE, cells were lysed in RIPA Buffer (Sigma, R0278) supplemented with complete protease inhibitor cocktail tablet (Roche), 5 mM β-glycerolphosphate, 5 mM NaF and 1 mM sodium orthovanadate. Protein quantification was performed with the DC Protein Assay kit (Bio-Rad). Protein samples were boiled in NuPAGE™ LDS Sample Buffer 4x (Novex, NP0007) with 10 mM DTT at 70°C and separated with 4–12% NuPage Bis–Tris gels (Novex, NP0321). Proteins were transferred to a PVDF membrane, subsequently blocked with 5% skimmed milk in PBST (PBS supplemented with 0.1% Tween-20) for 1 h at RT and thereafter incubated with primary antibodies overnight at 4°C. On the following day, membranes were washed in PBST, incubated with the appropriate HRP-conjugated secondary antibodies for 1 h at RT, then washed and developed using AmerSham™ ECL™ Western Blotting Detection Reagents (GE Healthcare, RPN2106). Chemiluminescence detection was done with an AmerSham™ Imager 600. The primary antibodies used were mouse monoclonal ATRX (Santa Cruz, sc-55584) and mouse vinculin (Sigma, V9131).

### Immunofluorescence and high content microscopy

For immunofluorescence (IF) assays, 5000 cells were seeded on μCLEAR 96-well plates (Greiner Bio-One, 655090) for high content imaging, or on coverslips. The cells were pre-extracted with ice cold 0.2% Triton X-100 (Sigma, T8787) in PBS for 1 min on ice and then fixed with 4% formaldehyde (VWR Chemicals) for 15 min at RT. For immunostaining of 53BP1/Cyclin-A, cells were directly fixed with 4% formaldehyde for 15 min RT and then permeabilized with 0.5% Triton X-100 in PBS for 10 min. Cells were washed and blocked with 2.5% BSA (Sigma, A7030) in PBST for 30 min and then incubated with primary antibody at 4°C overnight. On the following day, wells were washed in PBST, fluorescence-tagged secondary antibody (Alexa Fluor™ Goat Anti-mouse IgG 488 (Invitrogen, A11001); Alexa Fluor™ Chicken anti-Rabbit IgG 647 (Invitrogen, A21443)) added for 2 h at RT in the dark and stained with DAPI for 10 min. The primary antibodies used were ATRX (Santa Cruz, sc-55584), FANCD2 (Novus Biologicals, NB100-182), 53BP1 (Novus Biologicals, 100-304A2), Cyclin A (Abcam, ab16726) and DAXX (Bethyl Laboratories, A301-353A). For EdU (Life Technologies, A10044) incorporation analysis, EdU was added 30 min prior to fixing the cells and click chemistry done prior to blocking. A dilution of 10 mM ascorbic acid was prepared fresh, and the click-it reaction mix was performed by adding PBS, CuSO_4_, Az. 647 and ascorbic acid in given order which was then added to each well for 1 h RT in the dark. Images were acquired using an Olympus IX-83 or for high content imaging the ScanR acquisition software (Olympus) controlling a motorized Olympus IX-81 wide-field microscope. Olympus UPLSAPO 10×/0.4 NA and 40x/0.9 NA objectives were used. For counting of foci, z-stack images were acquired and the ScanR analysis software (Olympus) was used to count the total number of foci per cell.

### Preparation and analysis of chromosome spreads

HeLa cells seeded in 10-cm dishes were transfected with indicated siRNAs. Two days post transfection cells were treated with DMSO (Sigma, D2438) or APH (0.4 μM) (Sigma, A0781) for 20 h. Colcemid (0.1 μg/ml) (ThermoFisher Scientific, 15210-040) was added 5 h prior to collection of samples. Cells were harvested and collected in a 15 ml tube. After centrifugation for 5 min (300 x g), the supernatant was removed, the pellet was carefully resuspended and 5 ml of pre-warmed (37°C) 75 mM KCl (Sigma) was added drop-by-drop. The cell suspension was then incubated for 20 min at 37°C. Swollen cells were collected by centrifugation for 6 min (300 x g) and resuspended with 5 ml of fixation solution methanol:acetic acid (3:1) added drop-by-drop while vortexing carefully. Fixed cells were incubated at RT for 30 min followed by at least 1 h at –20°C. For preparation of chromosome spreads, cells were dropped on humid slides and air dried overnight. On the following day, slides were washed with PBS for 30 min and chromosomes stained using DAPI (Vectashield; Vector Labs). Images were captured using an Olympus BX63 microscope using 60X/OIL objective. The criteria for scoring breaks and gaps were as in (34).

### Analysis of MiDAS on chromosome spreads

HeLa cells seeded in 10-cm dishes were transfected with indicated siRNAs. Two days post transfection, cells were treated with DMSO (Sigma, D2438) or APH (0.4 μM) (Sigma, A0781) for 20 h and RO-3306 (7 μM) (Sigma, SML0569) was added for the last 6 h. Cells were subsequently washed twice with pre-warmed PBS, once with pre-warmed DMEM and released into media containing EdU (20 μM) and colcemid (0.1 μg/ml) for one more hour. Chromosome spreads were prepared as described in the section above. For EdU Click-IT reaction, slides were fixed with 4% formaldehyde for 11 min and blocked three times with PBS containing 4% BSA for 10 min. Subsequently, EdU detection was performed by using Click-IT Plus EdU Alexa Fluor 594 Imaging Kit following manufacturer's instructions (Thermo Fischer Scientific). Chromosomes were stained using DAPI (Vectashield; Vector Labs) and images were captured with an Olympus BX63 microscope using 60×/oil objective.

### Fluorescence in situ hybridization (FISH)

Chromosome spreads were prepared as described above. FRA16D FISH probe was prepared from the BAC clone RP11-264L1 (GenBank: AC046158.6) and labeled using the BioNick labeling system (Thermo Fisher Scientific). Chromosome spreads were dehydrated in an ethanol series (70%, 90%, 99%) at RT for 2 min each. Samples were denatured in 70% formamide at 75°C for 2.5 min and dehydrated again with ice-cold ethanol series (70%, 90%, 99%) for 2 min each. FISH probe was denatured at 93°C for 5 min and hybridization was performed at 37°C for 72 h. Biotin-conjugated probe was detected using avidin-FITC (Thermo Fisher Scientific) and biotin-labeled anti-avidin (Vector Labs). Chromosomes were stained using DAPI (Vectashield; Vector Labs) and images were captured with an Olympus BX63 microscope using 60×/oil objective.

### Immunofluorescence combined with FISH

For experiments where IF was combined with FISH, HeLa cells were treated with DMSO (Sigma, D2438) or APH (0.4 μM) (Sigma, A0781) for 20 h and 6 μM RO-3306 (Sigma, SML0569) was added for the last 6 h of treatment to arrest cells in G2. Cells were harvested with trypsin and seeded on slides using a Cytospin 4 cytocentrifuge (Thermo Fisher Scientific). Samples were pre-extracted and fixed with 4% formaldehyde (VWR Chemical) and 0.2% Triton X-100 (Sigma, T8787) in cold PBS for 20 min at 4°C. Slides were washed in PBS and ATRX IF was performed as described in the IF method section. Prior to FISH experiments, samples were re-fixed with 4% formaldehyde. For FISH, samples were dehydrated in an ethanol series (70%, 90%, 99%) at RT for 2 min each, and samples were denatured at 90°C for 5 min. Hybridization and detection was performed as described above. Cells were stained using DAPI (Vectashield; Vector Labs) and images were captured with an Olympus BX63 microscope using 60×/oil objective.

### Statistical analysis

Statistical analysis was performed using GraphPad Prism 7 using the unpaired t-test method for significance testing. To validate that ATRX and FANCD2 foci colocalization changes upon APH treatment were not product of chance we used the JACoP plugin for FIJI (35). To assess significance of FRA16D breakage in chromosome spreads we used the chi-square statistical test.

## RESULTS

### Quantitative MS-based approach for identification of CFS interacting proteins

**Figure 1.**
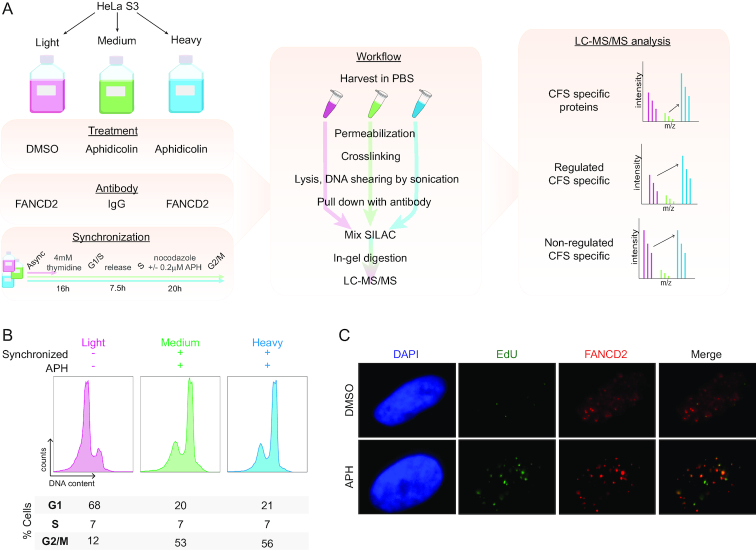
Workflow for MS-based quantification of CFS associated proteins. (**A**) Experimental workflow for SILAC-based quantitative MS identification of CFS associated proteins. (**B**) Flow cytometry analysis of cell cycle distribution of SILAC labeled cells used for enrichment of CFSs as illustrated in (A). (**C**) IF of FANCD2 and EdU incorporation to assess formation of FANCD2 foci at late replicating regions with and without APH treatments. Cells were synchronized as in (A) and (B).

To identify new regulators of CFS stability, we developed a workflow to perform global analysis of CFS binding proteins using SILAC-based quantitative MS (26). Our methodology is based on the enrichment of chromatin associated to FANCD2, a protein that has been described to localize specifically to CFS in G2/M cells (23) (Figure 1A). Critically, we used telomerase-positive HeLa S3 cells to avoid the enrichment of telomeric regions, as FANCD2 also localizes at telomeres in ALT-positive cell lines (36). To create conditions of mild RS that mimic those of oncogenic transformation and that cause CFS instability, we treated the cells with low doses of APH (0.2–0.4 μM). These conditions increase the number of challenged CFSs harboring FANCD2 in G2/M (23) and are typically used to study the mechanisms and proteins that resolve CFS aberrations (21,23,37,38). Additionally, to enhance the number of cells with FANCD2 localized at CFSs, cells were synchronized in G2/M. Briefly, cells were blocked at the G1/S boundary with thymidine for 16 h, followed by 7.5 h release into S phase and finally arrested at prometaphase by 20 h nocodazole treatment with or without APH to increase the number of challenged CFSs (Figure 1A). The synchronization efficiency was evaluated by cell cycle distribution analysis using flow cytometry, to confirm that indeed the majority of cells were in G2/M (Figure 1B). The cell cycle profiles obtained were similar in medium and heavy SILAC labeled cells, which were both synchronized and treated with APH, while the light-labeled cells were maintained asynchronous and without APH treatment (Figure 1B). We validated that, in accordance with the literature, the APH treatment exacerbated the number of FANCD2 foci in G2/M cells, and using EdU (5-Ethynyl-2′-deoxyuridine) as a marker of ongoing replication we confirmed that these foci were late replicating sites and thereby CFSs (23) (Figure 1C). Finally, to enrich proteins associated with these challenged CFSs, we performed a ChIP of endogenous FANCD2 after extraction of the soluble fraction by permeabilization and subsequent crosslinking with formaldehyde (Figure 1A). IgG was used for ChIP from the medium labeled condition to allow assessment of background interactors in the FANCD2 pull-down (Figure 1A).

After immunoprecipitation, formaldehyde crosslinks were reversed and proteins were separated by SDS-page followed by in-gel digestion prior to state-of-the-art nano liquid-chromatography tandem-MS (LC-MS/MS) analysis on a Q-Exactive HF-X orbitrap mass spectrometer (22). All raw LC–MS/MS files from two biological replicates were processed and analyzed together in the MaxQuant software suite (29) (www.maxquant.org) using the integrated database search engine, Andromeda, with a 1% false discovery rate at the peptide and protein levels (30). The database search was performed together with raw files from a pilot experiment where different cell cycle profiles were obtained from a separate synchronization protocol (Supplementary Figures S1A and B). Only the medium (+APH, IgG ChIP) and heavy (+APH, FANCD2 ChIP) conditions from the pilot experiment were used for some downstream analysis. With this workflow, we performed a global analysis of proteins associated with FANCD2-enriched CFSs.

### Global analysis of the CFS interactome

**Figure 2.**
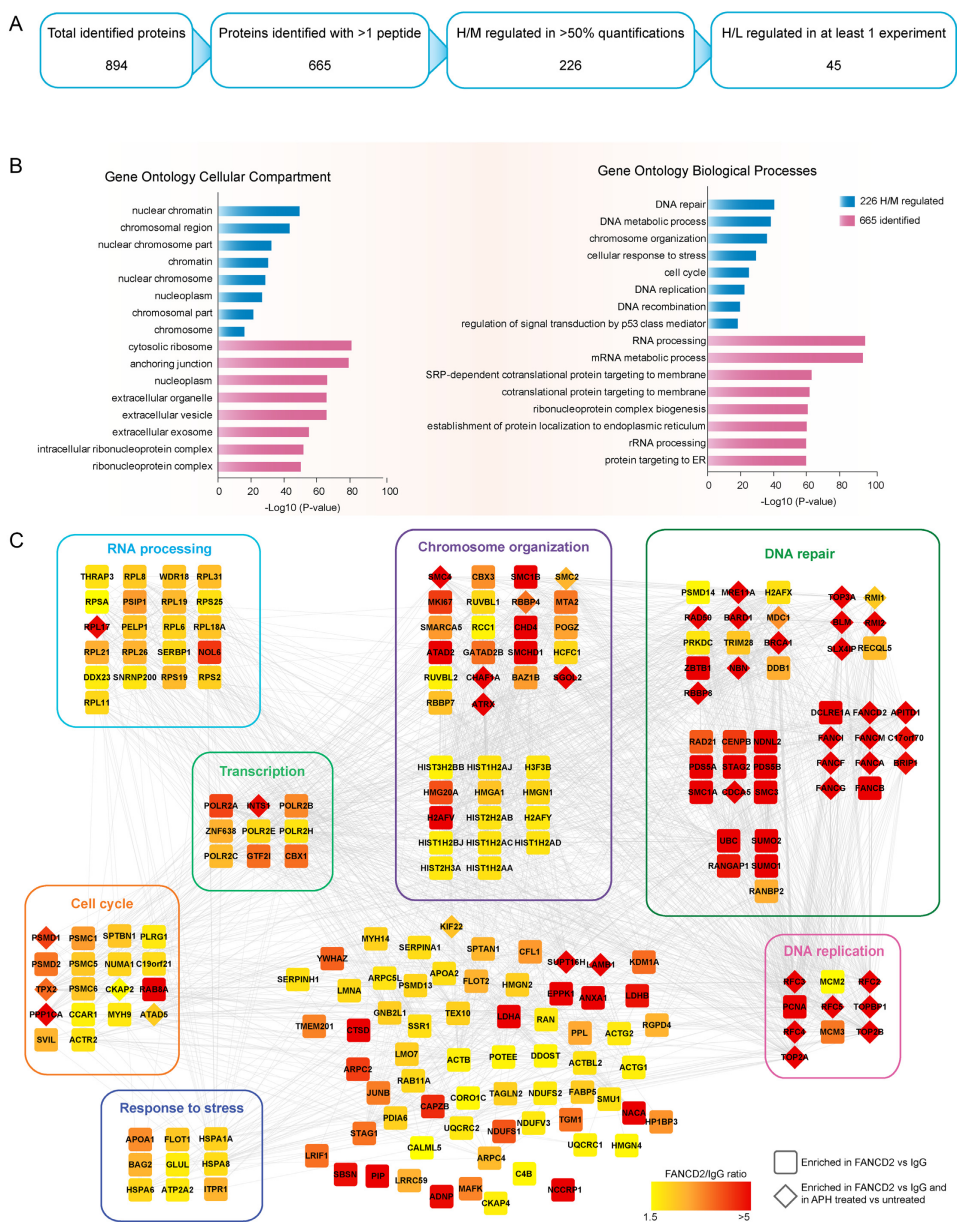
Overview and analysis of MS-based identification of CFS associated proteins. (**A**) Results and filtering of identifications from MS analysis. (**B**) GO term enrichment analysis for biological process and cellular localization within the 226 proteins that were more abundant in the FANCD2 pull-down compared to IgG. Analysis was performed using the DAVID tool with the human genome as reference. (**C**) Functional network analysis of all 226 FANCD2 enriched proteins. The color of the nodes indicates the level on enrichment in the FANCD2 pull-down compared to IgG. Diamond shaped nodes indicate those that were enriched in the FANCD2 pull-down from APH treated cells compared to vehicle treated. Proteins with well-defined relevant functions have been grouped. The network was generated using the STRING database in Cytoscape.

To discriminate background binders from proteins that are associated with CFSs in our MS-based workflow, we stratified the protein groups identified in the database search by applying quantitative filters and validating the results with bioinformatics tools. The database search identified 894 protein groups, of which 665 were identified with more than one peptide and used for downstream analysis (Figure 2A and Supplementary Table S1). For potential CFS interactors we further required at least 1.5-fold higher abundance in the FANCD2 pull-down compared to IgG from synchronized and APH treated cells in more than half of the experiments from which they were identified. With these criteria, we categorized 226 potential CFS interactors, with the bait itself, FANCD2, and its constitutive interactor FANCI among the most enriched in the FANCD2 IP compared to IgG (Supplementary Figure S2A and Table S2). To determine whether these 226 proteins represented a group of identifications relevant for CFS stability, we performed Gene Ontology (GO) enrichment analysis. We found that these proteins were primarily located at chromosome related compartments, and they were highly enriched for factors involved in DNA repair and chromosome organization as expected for CFS related proteins where these biological processes are central for maintaining CFS stability (Figure 2B).

To visualize the potential CFS interacting proteins, we performed a functional network analysis and grouped the proteins according to their known functions (Figure 2C). Notably, many proteins with known functions in maintenance of CFS stability were found to be highly enriched. Among these, FANCD2, FANCI and other proteins of the Fanconi Anemia complex were highly enriched, including APITD1, also known as CENPS, which promotes mono-ubiquitination of FANCD2-FANCI in response to DNA damage (39). We also found RBPP8, known as CtIP, and all the proteins of the MRN complex (Mre11, Rad50 and Nbs1) to be highly enriched. Importantly, the nuclease activities of Mre11 and CtIP have been shown to be required for DSB repair at CFSs (40). Furthermore, TOP3A, RMI1 and RMI2, which form the TRR complex that associates with BLM, and other proteins with well-established roles in the processing of challenged CFSs were found to be highly enriched (41–43). We also quantified a high enrichment of PCNA, components of the MCM helicase, and other replication proteins, which are associated with chromatin in mitotic cells upon RS (21), and would expectedly be enriched at CFSs with stalled replication forks. The data demonstrates that the proteomics-based workflow we developed, in combination with the analysis approach applied, is a powerful strategy to identify CFS associated proteins.

Notably, our data also showed a high enrichment of small ubiquitin like modifier (SUMO), a post-translational modification that is known to regulate protein functions at the chromatin in response to DNA damage and RS (44–47) (Figure 2C).

To date, the majority of the proteins involved in maintenance of CFS stability are established players in pathways contributing to safeguarding genomic stability, such as those responding to DSBs and RS. Notably, our data shows a strong enrichment for these categories of proteins, supporting that this data is a valuable resource to identify new regulators with a particular role in CFS maintenance.

### ATRX is associated with chromatin under conditions that induce CFS expression

Next, we identified the proteins that are recruited to challenged CFSs specifically upon perturbation of replication with APH. For this, we required that proteins deemed as potential CFS interactors (as defined above) were also enriched at least 1.5-fold in the FANCD2 pull-down from synchronized cells treated with APH (heavy) compared to untreated asynchronous cells (light) (Figure 2A). This resulted in a selective dataset of 45 proteins (diamond shaped nodes in Figure 2C and Supplementary Table S3), of which the top 20 are listed in Supplementary Figure S2. This subset of proteins is largely comprised of factors with known involvement in DNA repair, and proteins known to function in CFS stability, including FANCD2, FANCI, BLM and BRCA1 (Figure 2C) (16,20,43). Therefore, we consider that this dataset could also contain a number of proteins with uncharacterized functions at CFSs. Among these, we validated and characterized ATRX, a SNF-2 type chromatin remodeler, due to its previously defined functions and its known role as tumor suppressor (48). As such, ATRX has been shown to play an important role in telomere maintenance (49), regions which, similar to CFS, are notoriously difficult to replicate. Furthermore, ATRX has been implicated in the suppression of DNA–RNA hybrid (R-loop) stabilization at active transcription sites at telomeric repeats (50), an alteration that is also found at some CFSs as a consequence of replication-transcription collision.

**Figure 3.**
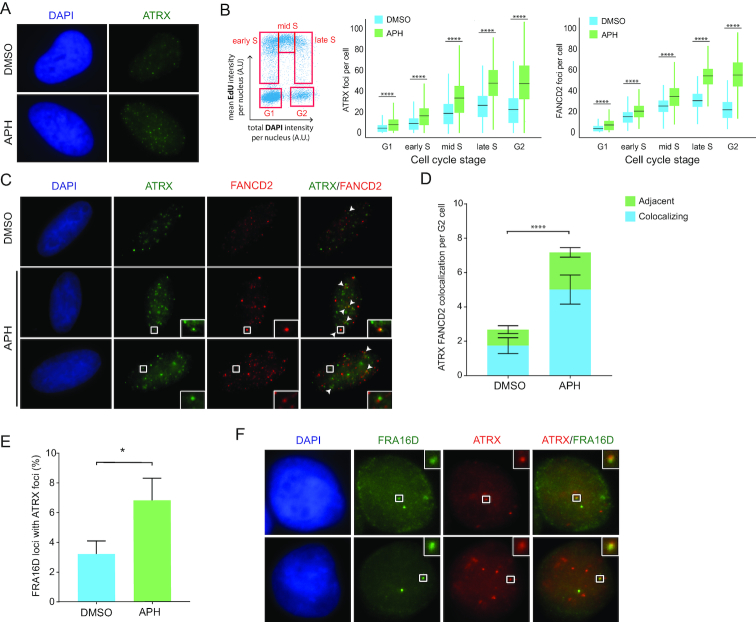
ATRX associates with chromatin under conditions that induce CFS expression. (**A**) IF analysis of ATRX (green) in G2 cells. HeLa cells were treated with DMSO or APH (0.2 μM) for 20 h, and G2 cells were selected based on DAPI profile. Representative images are shown. (**B**) Analysis of the number of ATRX and FANCD2 foci across the cell cycle. HeLa cells were incubated with DMSO or APH (0.2 μM) for 20 h and EdU (20 μM) was incorporated for the last 30 min. Analysis was performed using quantitative image-based cytometry (QIBC). DAPI and EdU profiles were used to determine cell cycle stages (G1, early, mid and late S and G2). Z-stack images were acquired to determine the number of foci per cell. The average number of ATRX foci per cell in each stage is illustrated in the box plots for vehicle (blue) or after APH treatment (green). Data shown corresponds to the average of >700 cells per condition. Means, SDs, maximum and minimum values are indicated. Significance was evaluated with unpaired *t*-test ****P ≤ 0.0001. (**C**) IF analysis for colocalization of ATRX (green) and FANCD2 (red) in G2 cells. HeLa cells were treated with DMSO or APH (0.2 μM) for 20 h. Two different colocalization patterns are indicated: complete foci overlap (middle row and indicated by white arrows) and adjacent foci (bottom row). Representative images are shown. Selected regions are magnified. (**D**) Quantification of FANCD2 and ATRX colocalizing foci per G2 cell. Data shown correspond to biological duplicates of 300 cells per condition. Means and SDs are indicated. Significance of the total number of colocalizing foci was evaluated with unpaired *t*-test ****P ≤ 0.0001. (**E**) Quantification of the percentage of FRA16D loci colocalizing with ATRX foci. HeLa cells were treated with DMSO or APH (0.2 μM) for 20 h and arrested at G2. Data shown corresponds to biological triplicates of 300 cells per condition. Means and SDs are indicated. Significance was evaluated with unpaired *t*-test. *P ≤ 0.05. (**F**) Example of ATRX IF (red) colocalizing with FRA16D (green) in G2 cells. Selected regions are magnified.

As a first indication for a potential role for ATRX in the maintenance of CFS stability, we assessed whether ATRX forms foci under conditions that provoke CFS instability (also known as CFS expression). To this end, we treated HeLa cells with a low dose of APH, and evaluated the formation of ATRX foci by IF combined with high-content microscopy (Figure 3A). We observed that ATRX forms discrete foci upon treatment with APH, and that this is increased compared to control cells, indicating that ATRX responds to perturbed replication (Figure 3A). Next, we sought to establish whether the formation of ATRX foci displayed the same pattern as FANCD2 foci, which peaks in late S and G2 where CFSs are replicating and therefore challenged, particularly upon APH treatment (Figure 3B). We treated cells with APH or vehicle, and used labeling with EdU and DAPI content to profile the different cell cycle stages (Figure 3B). Similar to FANCD2, the number of ATRX foci increased from G1 and across the cell cycle, peaking in late S and G2, a phenotype exacerbated at each stage upon treatment with APH (Figure 3B). Importantly, to confirm the specificity of ATRX signal obtained by IF, we knocked-down ATRX with two different siRNAs and observed an abrogation of the ATRX foci (Supplementary Figures S3A–C). A number of ATRX foci colocalized with FANCD2 in G2 cells upon APH treatment, where FANCD2 foci served as surrogate markers for CFSs (Figure 3C and D). Furthermore, the number of colocalizing foci increased upon mild RS with APH compared to untreated G2 cells, indicating that ATRX localizes to challenged CFSs (Figure 3C and D). We found that in addition to the fully colocalizing, a number of foci were adjacent to each other. As has been observed with other proteins that function in CFSs this pattern could arise due to different temporal stages of CFS processing (51). Interestingly, we observed that the percentage of FANCD2 foci colocalizing with ATRX was only mildly increased upon APH treatment, suggesting that the fraction of stressed CFSs at which ATRX functions is similar in these conditions (Supplementary Figure S3D). Of note, we could not detect a direct interaction between ATRX and FANCD2 by co-immunoprecipation experiments (data not shown).

Using the colocalization software *JACoP* and the Costes approach (35), we found that the Pearson coefficient for the colocalization of ATRX and FANCD2 was at least 10-fold higher in input G2 cell images compared to images of these same cells where the pixels had been randomized. These analyses indicate that the colocalization of ATRX and FANCD2 detected was not by chance with a probability higher than 99% (data not shown).

To further confirm that a subset of the ATRX foci in G2 is indeed located at CFSs, we performed FISH for FRA16D combined with ATRX-IF in G2 cells. We observed that ATRX localized to ∼3% of FRA16D loci in untreated G2 cells, increasing to 7% upon APH treatment (Figure 3E and F).

Together, these data indicate that ATRX is a novel protein that localizes to a percentage of challenged CFSs, and may therefore play a role in maintaining their stability.

### ATRX regulates CFS stability

**Figure 4.**
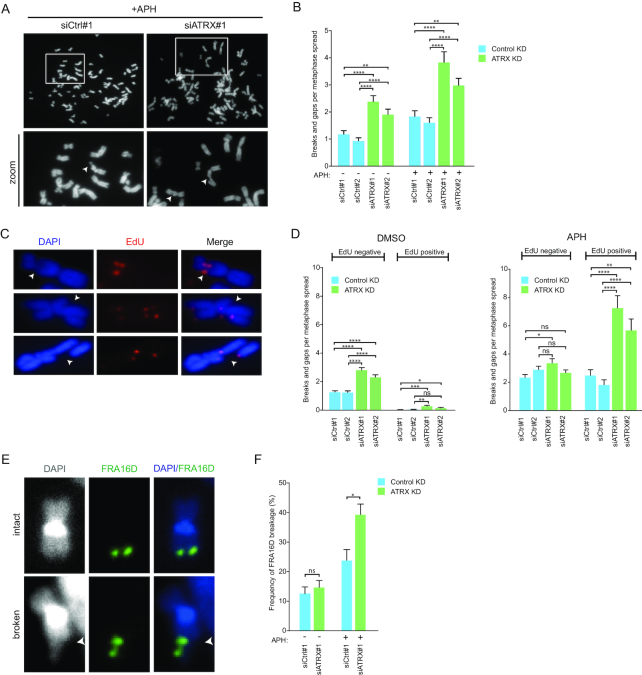
Effects of ATRX depletion on CFS stability. (**A**) Metaphase spreads of HeLa cells to assess CFS instability upon depletion of ATRX compared to transfection with control siRNAs. Two days post-transfection, cells were treated with DMSO or APH (0.4 μM) for 20 h. Representative images are shown. Arrows indicate representative gaps and breaks. (**B**) Quantification of gaps and breaks in metaphase spreads of HeLa cells transfected with indicated siRNA and treated as in (A). Average of 40 spreads per condition are shown. Mean and SEMs are indicated. Significance was evaluated with unpaired *t*-test **P ≤ 0.01;****P ≤ 0.0001. (**C**) Evaluation of MiDAS upon depletion of ATRX compared to transfection with control siRNAs. HeLa cells were treated with DMSO or APH (0.4 μM) for 20 h. Arrows indicate representative images of EdU-positive breaks. (**D**) Quantification of EdU-negative and -positive (MiDAS) gaps and breaks in metaphase spreads of HeLa cells transfected with the indicated siRNAs and treated as in (C). Data shown corresponds to the average of 60 spreads per condition from two biological replicates. Mean and SEMs are indicated. Significance was assessed with unpaired *t*-test. n.s. >0.05; *P ≤ 0.05; **P ≤ 0.01; ***P ≤ 0.001;****P ≤ 0.0001. (**E**) Evaluation of FRA16D fragility upon depletion of ATRX compared to transfection with control siRNA. HeLa cells were treated with APH (0.4 μM) or DMSO for 20 h. Representative images of intact and broken FRA16D are shown. (**F**) Quantification of the frequency of FRA16D fragility in metaphase spreads of HeLa cells transfected with the indicated siRNAs and treated as in (E). Data shown corresponds to biological triplicates of at least 120 FRA16D scored loci. Means and SDs are indicated. Significance was evaluated by Chi-square test. n.s. >0.05; *P ≤ 0.05. ATRX depletion was verified by WB or IF in all the experiments performed.

CFS expression is defined by the presence of chromosomal breaks or gaps in metaphase spreads from cells that have undergone replication in the presence of low dose of APH (14). Thus, to determine whether the localization of ATRX at a subset of CFSs plays a role in maintaining CFS stability, we evaluated the impact of ATRX depletion on chromosome integrity by studying metaphase spreads. We observed a significant increase in the number of chromosomal gaps and breaks upon depletion of ATRX with two separate siRNAs in comparison to control cells (Figure 4A, B and Supplementary Figure S4A). Notably, this increase was observed in normally proliferating cells, and was exacerbated under treatment with low dose of APH (Figure 4B), suggesting a role for ATRX in regulating CFS stability.

To confirm that these sites of breakage were CFSs, we assessed whether these regions were sites of active replication in mitosis, as it has been shown that CFSs undergo mitotic DNA synthesis (MiDAS) before completing mitosis (21). To this end, we evaluated the number of chromosomal gaps and breaks that incorporated EdU in metaphase spreads, in the absence and presence of low dose APH (Figure 4C, D and Supplementary Figure S4B). In accordance with previous studies, the majority of gaps and breaks were EdU negative in untreated conditions (21) (Figure 4D). However, we observed that ATRX knock down (KD) increased the number of EdU positive (MiDAS positive) gaps and breaks (Figure 4D), and that APH treatment resulted in a marked increase in this phenotype in comparison to ATRX proficient cells (Figure 4D). Finally, we investigated the effects of ATRX deficiency specifically on FRA16D, one of the CFSs with the highest propensity to break. We observed that depletion of ATRX in untreated conditions led only to a mild increase in FRA16D fragility, while addition of low dose of APH resulted in a significant increase in FRA16D fragility (Figure 4E and F). Together these data indicate that the increased breaks and gaps observed in metaphase spreads upon ATRX depletion occur at CFSs and that ATRX is important for maintaining CFS stability.

**Figure 5.**
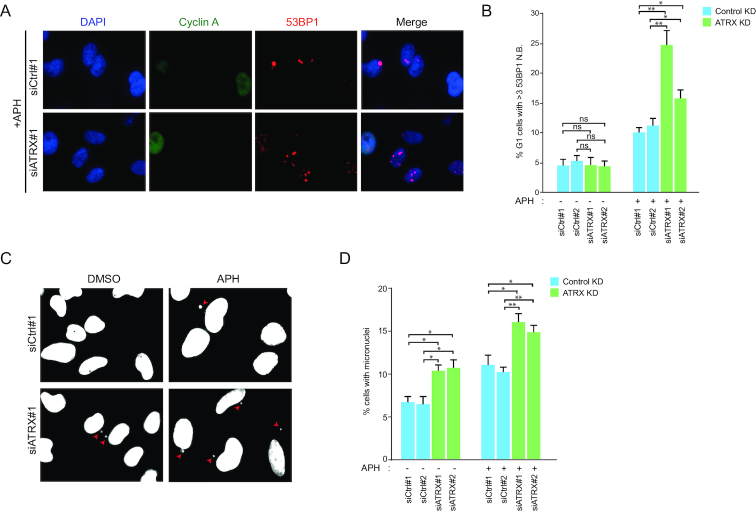
Consequences of ATRX depletion on markers of genomic instability. (**A**) 53BP1 N.B. in G1 cells were determined by Cyclin A (green) and 53BP1 (red) IF stainings in HeLa cells transfected with the indicated siRNAs. Two days post-transfection, cells were treated with DMSO or APH (0.2 μM) for 20 h. (**B**) Quantification of analysis from (A). Cyclin A negative cells were selected as G1 cells. Data correspond to biological duplicates of >200 cells per condition. Means and SDs are indicated. Significance was assessed by unpaired *t*-test. n.s *P* > 0.05; *P ≤ 0.05; **P ≤ 0.01. (**C**) Evaluation of micronuclei formation. HeLa cells were transfected with the indicated siRNAs. Two days post-transfection, cells were treated with DMSO or APH (0.2 μM) for 20 h. Red arrows indicate micronuclei in representative images. (**D**) Quantification of cells with micronuclei as described in (C). Data correspond to biological triplicates of >500 cells per condition. Means and SDs are indicated. Significance was assessed by unpaired *t*-test. *P ≤ 0.05; **P ≤ 0.01. ATRX depletion was verified by WB or IF in all the experiments performed.

Next, we investigated the cellular consequences of ATRX deficiency in relation to CFS instability. First, we observed that ATRX depletion combined with APH increased the number of cells with multiple 53BP1 nuclear bodies (N.B.) in G1 cells (Figure 5A and B). These nuclear structures are associated with CFS instability (52), and their induction suggests that the CFS instability provoked by ATRX depletion remains unresolved upon entry into the next cell cycle. Remarkably, we observed that ATRX frequently localizes to 53BP1 N.B. in G1, which supports a potential role of ATRX in modulating the repair of these genomic sites (Supplementary Figures S5A and B). In concordance with the above, ATRX KD led to an increase in the number of micronuclei, a phenotype that was also exacerbated by APH treatment (Figure 5C and D), further substantiating the importance of ATRX to maintain genomic stability. Together, these results reveal that, while ATRX depletion alone renders a mild increase in CFS instability, its absence renders a dramatic increase in CFS fragility upon replication perturbation, indicating that the mechanisms to maintain CFSs are compromised.

### ATRX functions with DAXX in CFS stability maintenance

Our data indicates that ATRX plays a role in CFS stability maintenance. However, the observed increase in CFS expression is most commonly a consequence of increased RS during S-phase. To address whether ATRX deficiency induces RS, we assessed the rate of EdU incorporation in ATRX KD cells, in the presence and absence of APH. We observed that DNA replication was unperturbed in ATRX depleted cells, and that the cell cycle profile was also unaffected (Supplementary Figures S5C and D), suggesting that the impact of ATRX deficiency on CFS stability does not occur due to a global increase of RS. Furthermore, the number of FANCD2 foci in G2 was unchanged upon ATRX KD, in normal conditions or upon RS (Supplementary Figures S6A and S6B), indicating that ATRX depletion does not lead to a higher number of stressed CFSs. Together, our data indicate that ATRX is not affecting the number of stressed CFSs and suggest that, instead, ATRX plays a role in the resolution of challenged CFSs.

**Figure 6.**
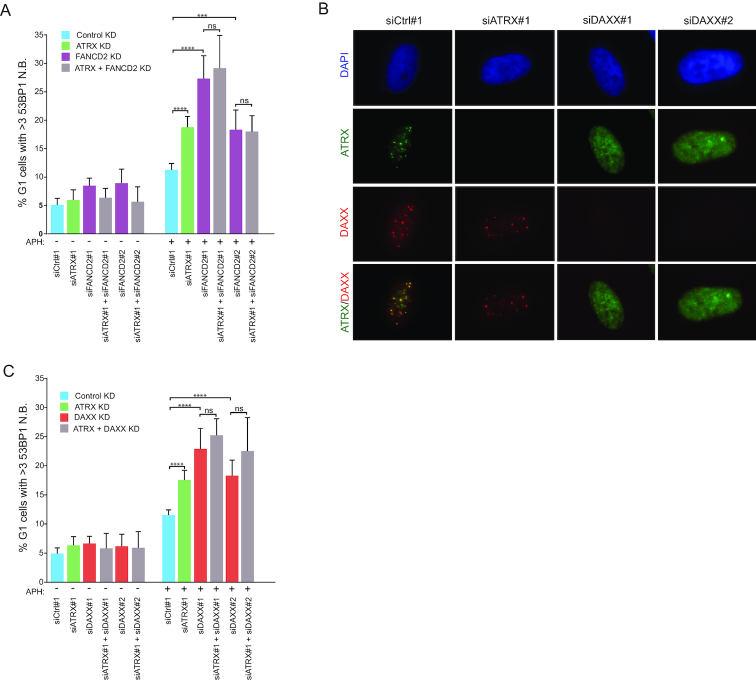
ATRX regulates CFS stability in a DAXX-dependent manner. (**A**) Quantification of 53BP1 N.B. in HeLa cells transfected with the indicated siRNAs. Two days post-transfection, cells were treated with DMSO or APH (0.2 μM) for 20 h. Cyclin A negative cells were selected as G1 cells. Data correspond to three biological replicates of >600 cells per condition. Means and SDs are indicated. Significance was assessed by unpaired *t*-test. n.s. >0.05;***P ≤ 0.001;****P ≤ 0.0001. (**B**) IF analysis of ATRX (green) and DAXX (red) in G2 cells. HeLa cells were transfected with the indicated siRNAs and two days post-transfection, cells were treated with APH (0.2 μM) for 20 h. Representative images are shown. (C) Quantification of 53BP1 N.B. in HeLa cells transfected with the indicated siRNAs. Two days post-transfection, cells were treated with DMSO or APH (0.2 μM) for 20 h. Cyclin A negative cells were selected as G1 cells. Data correspond to biological triplicates of >600 cells per condition. Means and SDs are indicated. Significance was assessed by unpaired *t*-test. n.s. >0.05;****P ≤ 0.0001. ATRX, FANCD2 and DAXX depletion was verified by IF in all the experiments performed.

To elaborate on this, we assessed the influence of FANCD2 on the role of ATRX in CFS stability. FANCD2 depletion resulted in a reduced number of ATRX foci in G2 cells, suggesting that formation of ATRX foci is partially dependent on FANCD2 (Supplementary Figure S6C–E). Furthermore, we observed that the number of 53BP1 N.B. in G1 induced by APH upon depletion of ATRX and FANCD2 together was not exacerbated compared to depletion of FANCD2 alone (Figure 6A and Supplementary Figure S6F). These results suggest that ATRX and FANCD2 function within a common pathway to limit CFS fragility, with FANCD2 plausibly functioning upstream of ATRX.

ATRX, together with DAXX, forms a chromatin remodeling complex that facilitates DNA repair processes, such as homologous recombination (53). We therefore sought to establish whether the role of ATRX at CFSs requires DAXX. Interestingly, DAXX formed foci upon treatment with low dose of APH, which displayed complete colocalization with ATRX foci (Supplementary Figure S6G). Moreover, the formation of DAXX foci across the cell cycle highly resembled the pattern observed for ATRX and FANCD2, being higher in APH treatment compared to control at all stages of the cell cycle, with the peak number of foci in late S and G2 (Supplementary Figure S6H and 3B). To determine whether this translates to a role for DAXX in the function of ATRX in CFSs, we first assessed the capacity to form foci for either factor in the absence of the other. Depletion of DAXX resulted in complete abrogation of ATRX foci formation upon APH treatment, while depletion of ATRX did not impair the formation of DAXX foci, suggesting that DAXX is required for ATRX recruitment to CFSs (Figure 6B). Accordingly, depletion of DAXX induced 53BP1 N.B. in G1 in response to APH, similar to ATRX KD, whereas additional depletion of ATRX did not exacerbate this phenotype (Figure 6C and Supplementary Figure S6I). Together, these results indicate that the function of ATRX in regulating CFS stability is dependent on DAXX.

## DISCUSSION

The repercussion of CFS fragility on genomic stability renders these regions of indisputable importance in human health and disease. It has become evident that cells have developed complex mechanisms to maintain CFS integrity. In this study, we performed the first quantitative MS-based global analysis to identify proteins recruited to CFSs under conditions when they are prone to breakage. With our strategy, we were able to quantify abundance changes of numerous proteins with known functions in CFS maintenance. We also identified other factors which, to date, have not been implicated in CFS integrity. Among those, we validated and characterized the function of the chromatin remodeler ATRX at CFSs. Our study revealed that ATRX indeed is critical for CFS stability, rendering this resource valuable to identify new proteins that safeguard these crucial genomic regions.

We identified over 200 potential CFS interacting proteins in our MS screen. Among the most enriched were FANCD2 and its constitutive interactor FANCI, thus validating our method. Interestingly, we also identified many proteins from the Fanconi Anemia core complex, which are important for the activity of the FANCD2–FANCI complex upon DNA damage (20). In accordance with current models of CFS maintenance, bioinformatics analysis indicated that our dataset contained a large subset of proteins involved in DNA repair, chromosome organization and DNA replication. Indeed, proteins involved in DNA repair were among the most highly enriched, many of which have already been reported to participate in CFS repair, including the MRN complex (MRE11, RAD50 and NBN), BRCA1 and its constitutive partner BARD1, and the BTR complex (BLM, TOP3A and RMI proteins) (16,40–43). These results validate our strategy to identify novel genome caretakers that remain to be characterized within the context of CFS stability.

Among the novel factors identified in our CFS proteome, we focused on the validation and characterization of ATRX. ATRX has been involved in several genome maintenance and repair pathways by which it may contribute to maintain CFS stability. First, ATRX has been reported to function in the resolution of R-loops at telomeres (50). Given that replication-transcription collision comprises a risk factor for CFS stability, this could be one of the mechanisms by which ATRX deficiency leads to increased CFS fragility. Second, it has been shown that upon laser irradiation induced DSBs, the chromatin remodeling complex ATRX/DAXX deposits H3.3 to facilitate homologous recombination, an important pathway that regulates CFS stability (53,54). Finally, a recent study shows that ATRX deficiency promotes G-quadruplexes stabilization and DNA damage in glioblastoma cells (55). CFSs are regions prone to form secondary structures; therefore G-quadruplexes stabilization caused by ATRX deficiency could also contribute to CFS instability. In this study, we found that ATRX, together with DAXX, is indeed required for CFS stability, and particularly when the integrity of these regions is challenged by RS. We observed that ATRX localizes to some CFSs together with FANCD2 in G2 cells, and that this phenotype is increased upon conditions that challenge CFS stability, such as mild RS upon treatment with a low dose of APH.

Induction of RS could indeed account for the increased CFS fragility. In this regard, studies have found that ATRX may play a role in the RS response, since knock-out (KO) cells have shown decreased viability and DNA replication (56). However, in our system, loss of ATRX did not lead to increased RS or perturbed the cell cycle upon efficient transient depletions. Importantly, we did not observe an increase in FANCD2 foci upon ATRX depletion. This suggests that ATRX does not directly increase RS and does not contribute to induction of CFS stress. It is tempting to speculate that the accumulation of genomic alterations due to CFS instability over several cell cycles may cause the more severe phenotypes observed in ATRX KO cells such as replication defects and decreased viability (56).

However, the increased incidence of DNA breaks, and other hallmarks of CFS instability, such as MiDAS, 53BP1 N.B. and micronuclei upon depletion of ATRX indicates that this protein functions in the downstream repair of damaged CFSs. Indeed, our data indicates that, when present at the same CFS, ATRX and FANCD2 probably function within the same pathway that attempt to repair stressed CFSs prior to mitotic entry. Specifically, the reduction of ATRX foci upon FANCD2 depletion and lack of exacerbated phenotype upon double depletion of these two factors, indicate that ATRX may function downstream of FANCD2.

Unlike FANCD2, however, ATRX only functions in a subset of stressed CFSs, presumably those undergoing a particular repair process for which ATRX is necessary. This is evident with the percentage of FANCD2 foci colocalizing with ATRX which is around 10%. Our data indicate that ATRX regulates CFS stability in a DAXX-dependent manner, which may favor the view that the main molecular mechanism involved is the deposition of H3.3 histones to facilitate DSB repair by homologous recombination. However, it remains to be clarified whether the role of ATRX limiting R-loops or G-quadruplexes is DAXX-dependent.

Compromised ATRX function is the underlying cause of a rare neurodevelopmental syndrome (Alpha thalassemia retarded X-linked), a phenotype also typical of other disorders associated with increased fragile site stability, including Jacobsen syndrome (increased FRA11B breakage) (57). On the other hand, mutations in ATRX are strongly associated with pediatric glioblastoma (58). Importantly, our data places CFS instability as a potential source of genomic instability that could partially explain these and other pathologies associated to ATRX deficiency.

Here, we have presented a global proteomics analysis of CFSs and the identification of ATRX as a novel regulator of CFS stability. We believe that the presented proteomics characterization of CFSs provides a valuable resource to identify new proteins that function in the maintenance of these poorly understood regions of the human genome.

## DATA AVAILABILITY

All raw MS data for this study have been deposited to the ProteomeXchange Consortium via the PRIDE partner repository with the dataset identifier PXD011937.

## Supplementary Material

gkz510_Supplemental_Files
