# Proteomic characterization of chromosomal common fragile site (CFS)-associated proteins uncovers ATRX as a regulator of CFS stability

**DOI:** 10.1093/nar/gkz591

**Published:** 2019-07-03

**Authors:** David Pladevall-Morera, Stephanie Munk, Andreas Ingham, Lorenza Garribba, Eliene Albers, Ying Liu, Jesper V Olsen, Andres J Lopez-Contreras

**Affiliations:** 1 Department of Cellular and Molecular Medicine, Center for Chromosome Stability and Center for Healthy Aging, University of Copenhagen, Copenhagen 2200, Denmark; 2 Proteomics Program, Novo Nordisk Foundation Center for Protein Research, Faculty of Health and Medical Sciences, University of Copenhagen, 2200 Copenhagen, Denmark

*Nucleic Acids Research*, 2019, gkz510, https://doi.org/10.1093/nar/gkz510

The Authors wish to correct the BAC clone and GenBank access number used to prepare the FRA16D FISH probe from:

FRA16D FISH probe was prepared from the BAC clone RP11-383P16 (GenBank: AC233288.1) and labeled using the BioNick labeling system (Thermo Fisher Scientific).

To:

FRA16D FISH probe was prepared from the BAC clone RP11-264L1 (GenBank: AC046158.6) and labeled using the BioNick labeling system (Thermo Fisher Scientific).

The published article has been updated. This correction does not affect the results or conclusion of the article.

