# Supplementary material for: Proteomic characterization of chromosomal common fragile site (CFS)-associated proteins uncovers ATRX as a regulator of CFS stability
Source: Nucleic Acids Res. 2019 Jun 10;47(15):8004–18. doi: 10.1093/nar/gkz510 (PMC6735892; doi:10.1093/nar/gkz510)
Supplement: gkz510_Supplemental_Files [file gkz510_supplemental_files.zip › Supplementary data.pdf]

Figure S1

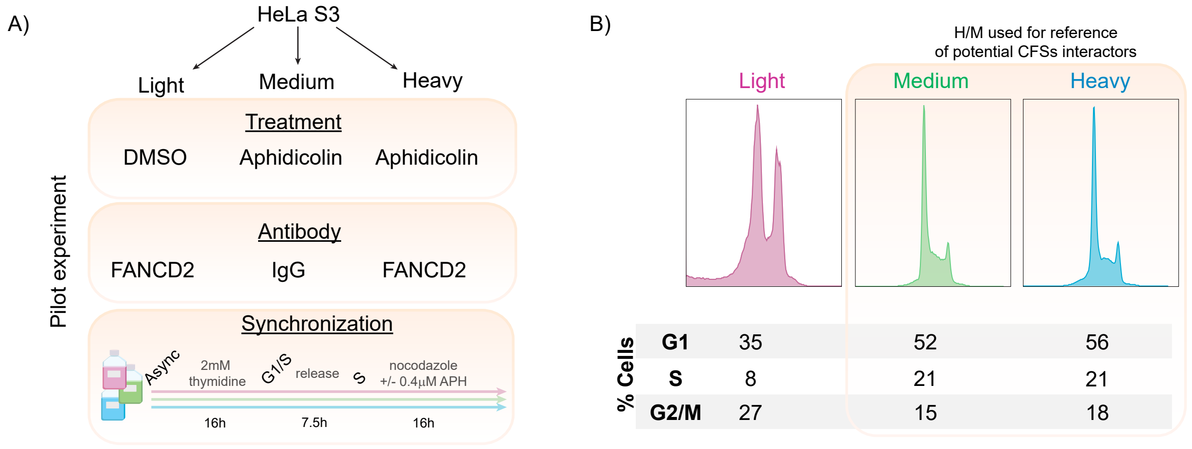

Figure S1 A) Experimental setup for MS analysis of FANCD2 associated chromatin bound proteins for pilot experiment. B) Flow cytometry analysis of cell cycle profile for pilot experiment. From this experiment only the heavy and medium conditions were used for the identification of potential CFSs interactors. Related to Figure 1.

Figure S2

A)

Protein names	Gene names	Uniprot	Peptides	Sequence coverage [%]	APH vs UT*	FANCD2 vs IgG**
Fanconi anemia group I protein	FANCI	Q9NVI1	82	55.00	2.57	>10.00
Fanconi anemia group D2 protein	FANCD2	Q9BXW9	59	39.70	2.53	>10.00
Structural maintenance of chromosomes protein	SMC4	Q9NTJ3	2	1.70	3.75	>10.00
DNA topoisomerase 2-binding protein 1	TOPBP1	Q92547	2	1.60	2.70	7.61
Shugoshin-like 2	SGOL2	Q562F6	12	13.60	3.64	6.18
Double-strand break repair protein MRE11A	MRE11A	P49959	9	14.30	2.51	5.98
Laminin subunit beta-1	LAMB1	P07942	2	1.20	6.07	5.59
Sororin	CDCA5	Q96FF9	3	12.40	4.40	5.57
DNA repair protein RAD50	RAD50	Q92878	21	16.10	2.33	5.06
Integrator complex subunit 1	INTS1	Q8N201	2	0.90	2.97	4.84
DNA topoisomerase 2-alpha	TOP2A	P11388	54	34.90	3.18	4.81
Chromatin assembly factor 1 subunit A	CHAF1A	Q13111	2	3.70	4.89	4.81
Transcriptional regulator ATRX	ATRX	P46100	3	1.40	2.44	4.73
DNA topoisomerase 2	TOP2B	Q02880	22	18.30	2.29	4.43
Targeting protein for Xklp2	TPX2	Q9ULW0	15	23.30	3.86	3.26
Mediator of DNA damage checkpoint protein 1	MDC1	Q14676	2	2.90	3.11	3.20
Structural maintenance of chromosomes protein 2	SMC2	O95347	3	4.20	2.86	2.71
Kinesin-like protein KIF22	KIF22	Q14807	4	7.90	3.03	2.43
ATPase family AAA domain-containing protein 5	ATAD5	Q96QE3	5	5.40	3.14	2.33
Cytoskeleton-associated protein 2	CKAP2	Q8WWK9	2	6.10	2.85	1.59

* Average H/L ratio for the two biological replicates. Note: APH is G2/M synchronized while the untreated is asyncrhonous.
** Average of all four biological replicates comparing FANCD2 and IgG pull downs in synchronized and aphidicolin treated cells. Both condiitons were G2/M synchronized.

Figure S2 A) List of the 20 most enriched proteins from the APH treated compared to untreated FANCD2 pull-down (H/L) (based only on proteins that were enriched in the APH FANCD2 pull-down compared to IgG(H/M)). Potential CFS interactors were categorized as proteins that were enriched in FANCD2 pulldown compared to IgG in synchronized and APH treated cells (H/M), in more than half the experiments in which they were quantified. For categorization of stress enriched CFS interactors, H/M enriched proteins were required to be regulated in APH treated synchronized cells vs untreated asynchronous cells (H/L) in at least one replicate. Related to Figure 2.

Figure S3

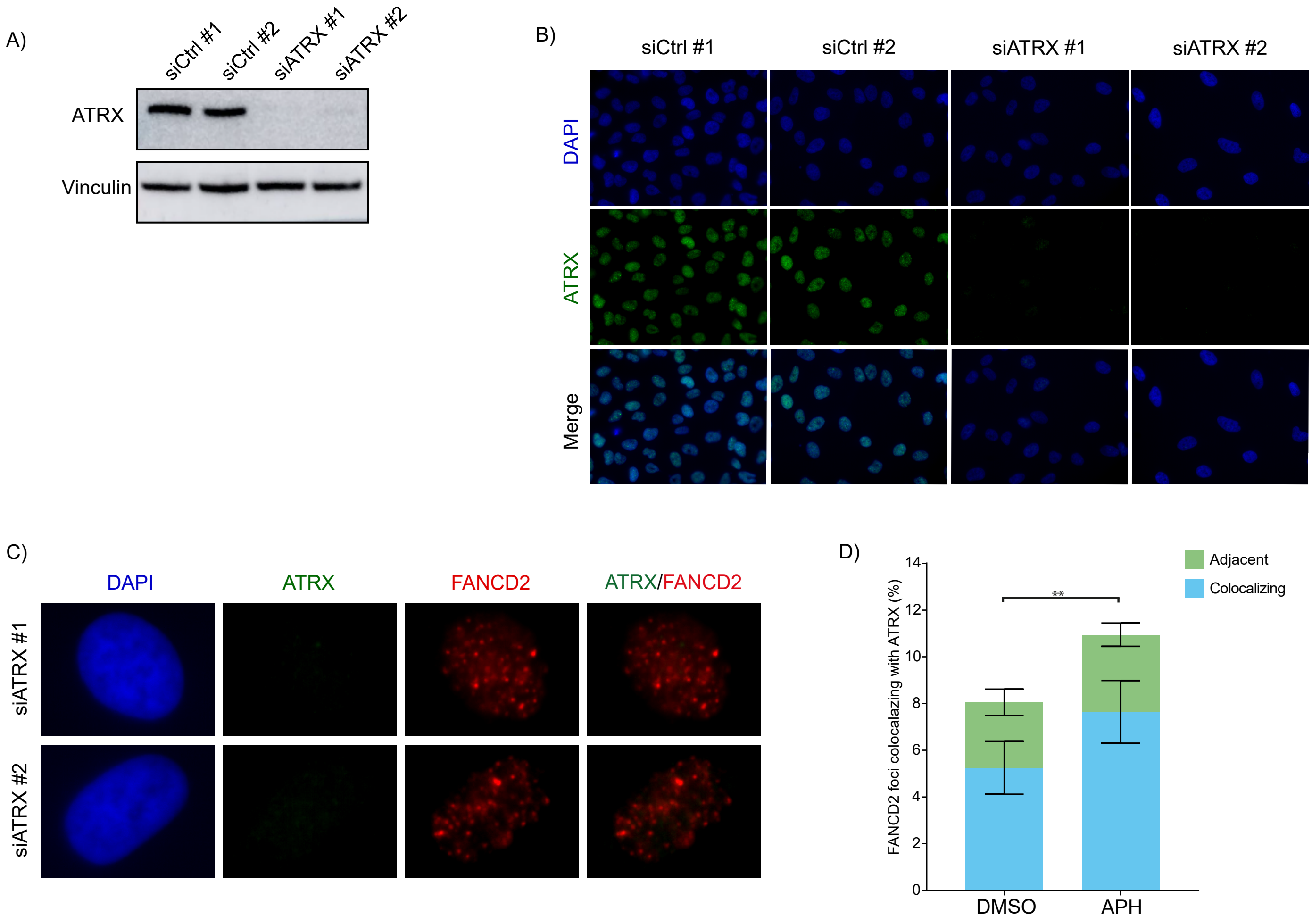

Figure S3 A) Analysis of ATRX KD efficiency. Western blots analysis of whole cells extracts from HeLa cells transfected with indicated siRNAs. B) Assessment of ATRX knockdown efficiency by IF. Representative pictures of ATRX IF stainings (green) in HeLa cells transfected with the indicated siRNAs. C) Assessment of ATRX antibody specificity in ATRX depleted cells. Representative pictures of IF staining of ATRX (green) and FANCD2 (red) in HeLa cells transfected with the indicated siRNAs. D) The percentage of FANCD2 foci colocalizing with ATRX out of total FANCD2 foci per G2 cell. Data shown corresponds to biological duplicates of 300 cells per condition. Means and SDs are indicated. Significance was evaluated with unpaired t-test. **p≤0.001. Related to Figure 3.

Figure S4

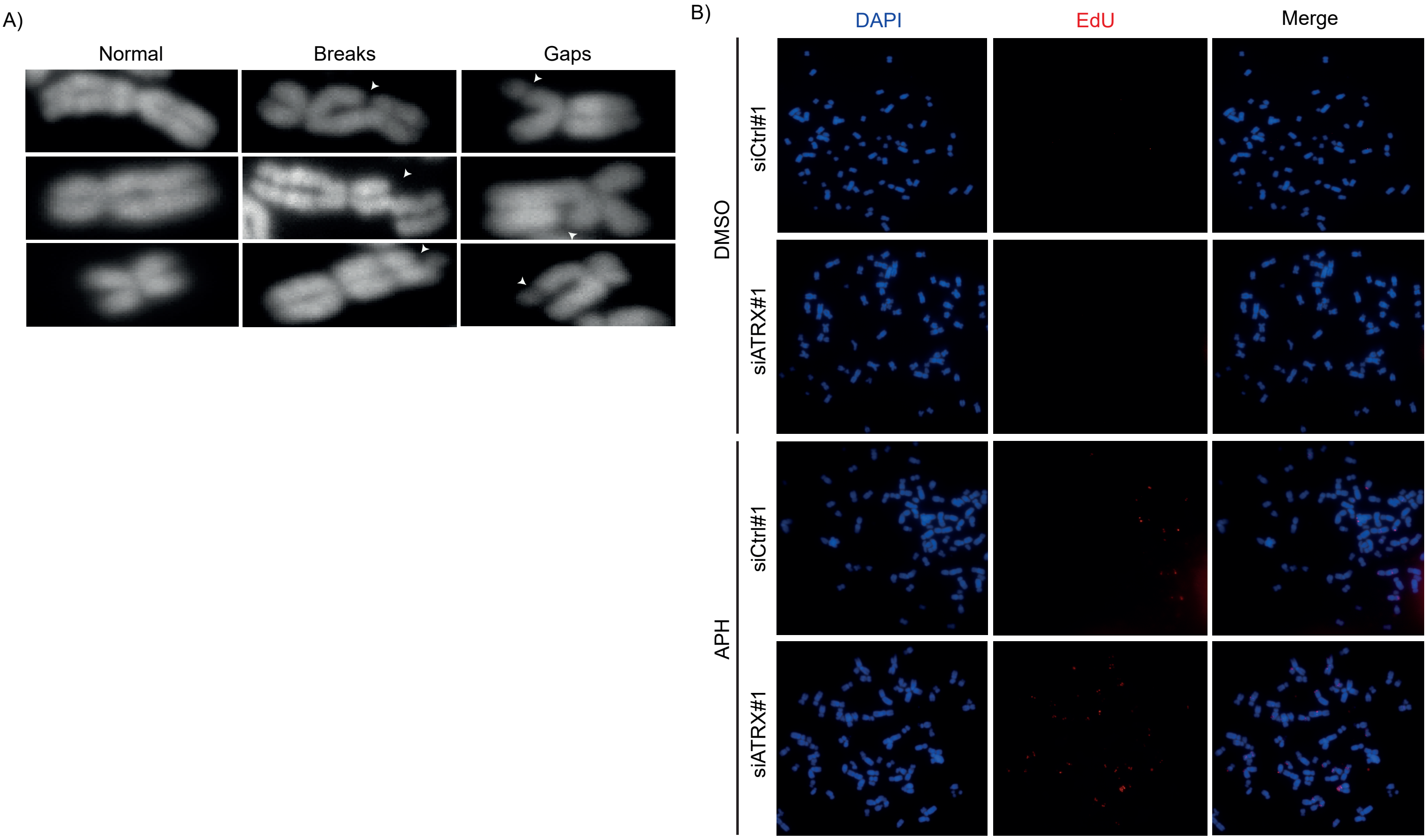

Figure S4 A) Examples of intact HeLa chromosomes and chromosomes harboring gaps or breaks (indicated by white arrows) as used for analysis of metaphase spreads in Figure 4. B) Representative images of MiDAs related EdU incorporation (red) on chromosome spreads (blue). HeLa cells were transfected with the indicated siRNAs. Two days post-transfection cells were treated with DMSO or APH (0.4 µM) for 20h. RO-3306 (7 µM) was added for the last 6 hours (late G2 arrest) and released into fresh medium containing EdU (20 µM) and colcemid (0.1 µg/mL) for further 60 minutes before collection. Related to Figure 4.

Figure S5

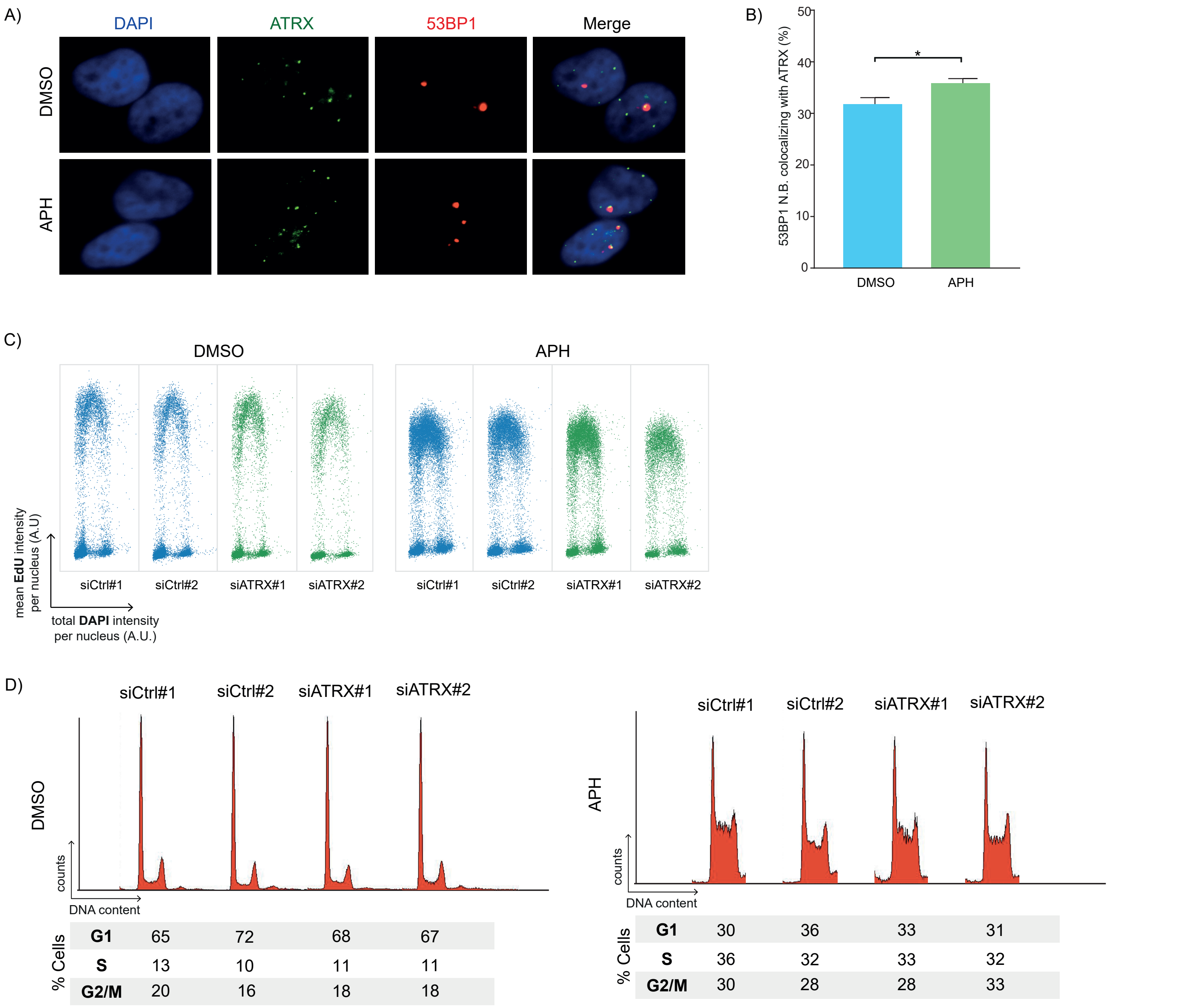

DMSO (% Cells)

	siCtrl#1	siCtrl#2	siATRX#1	siATRX#2
**G1**	65	72	68	67
**S**	13	10	11	11
**G2/M**	20	16	18	18

APH (% Cells)

	siCtrl#1	siCtrl#2	siATRX#1	siATRX#2
**G1**	30	36	33	31
**S**	36	32	33	32
**G2/M**	30	28	28	33

Figure S5 A) Representative pictures of IF staining of ATRX (green) and 53BP1 (red). HeLa cells were treated with DMSO or APH (0.2 μM) for 20h. G1 cells were selected by DAPI content. B) Quantification of the percentage of 53BP1 N.B. colocalizing with ATRX foci in G1 cells and treated as in A). Data shown corresponds to >900 cells per condition of three biological replicates. Means and SDs are indicated. Significance was evaluated with unpaired t-test. *p≤0.05. C) Quantitative image-based cytometry (QIBC) of HeLa cells was performed and total DAPI/mean EdU intensities were plotted in scatter diagrams showing replication profiles. HeLa cells were transfected with the indicated siRNAs and two days post-transfection cells were treated with DMSO or APH (0.2 μM) for 20 h. D) Flow cytometry analysis of cell cycle profile of HeLa cells transfected with the indicated siRNAs. Two days post-transfection cells were treated with DMSO or APH (0.2 μM) for 20 h. Related to Figure 5.

Figure S6

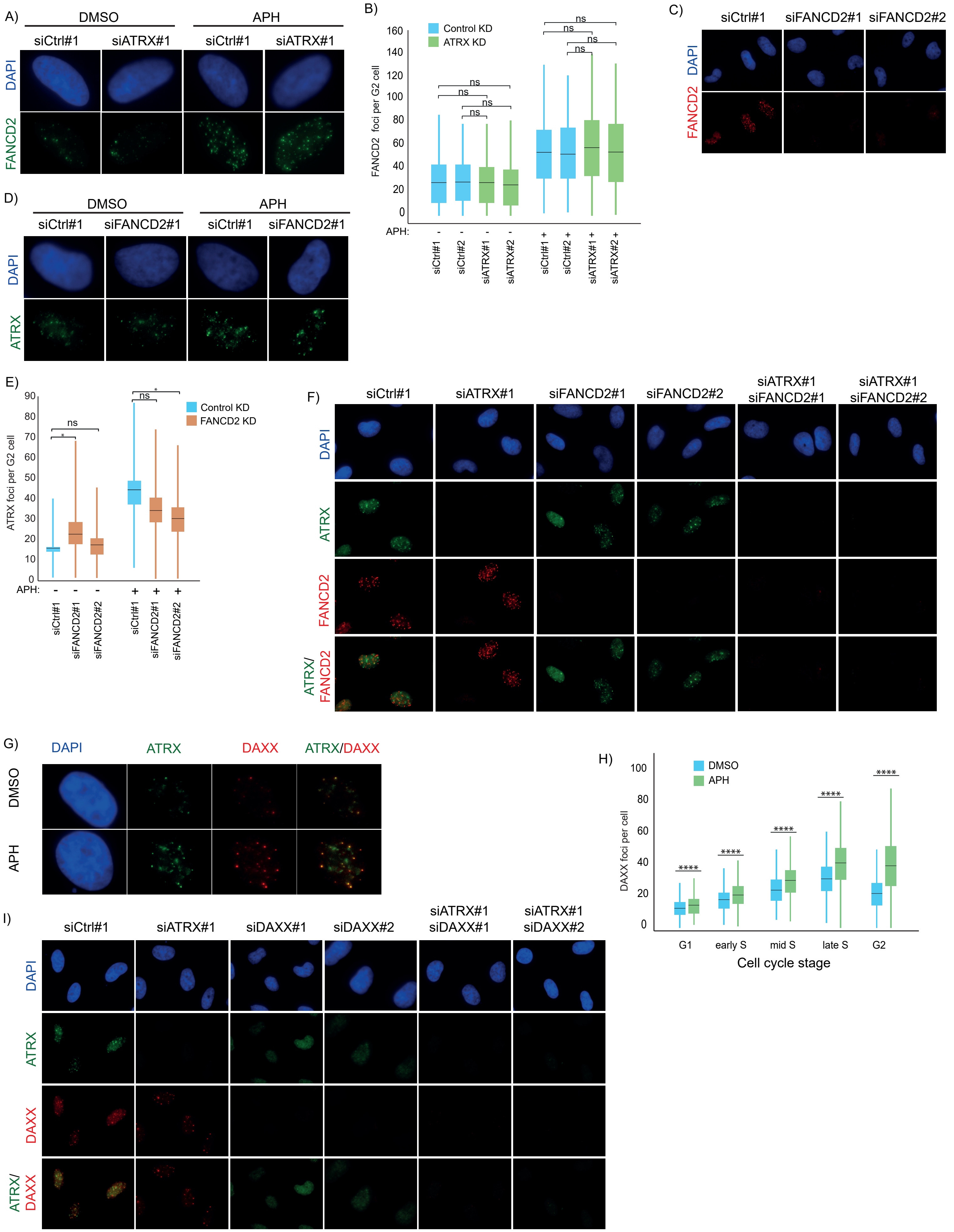
A)
DMSO
APH
siCtrl#1
siATRX#1
DAPI
FANCD2
B)
Control KD
ATRX KD
FANCD2 foci per G2 cell
ns
APH:
siCtrl#1
siCtrl#2
siATRX#1
siATRX#2
C)
siCtrl#1
siFANCD2#1
siFANCD2#2
D)
ATRX
E)
ATRX foci per G2 cell
FANCD2 KD
F)
siATRX#1 siFANCD2#1
siATRX#1 siFANCD2#2
ATRX/ FANCD2
G)
DAXX
ATRX/DAXX
H)
DAXX foci per cell
Cell cycle stage
G1
early S
mid S
late S
G2
****
I)
siDAXX#1
siDAXX#2
siATRX#1 siDAXX#1
siATRX#1 siDAXX#2
ATRX/ DAXX

Figure S6 A) IF analysis of FANCD2 (green) in G2 HeLa cells transfected with the indicated siRNAs. Two days post-transfection cells were treated with DMSO or APH (0.2 μM) for 20h. B) HeLa cells were transfected with the indicated siRNAs and treated as in A). Box plots show the average of FANCD2 foci per G2 cell. Data shown corresponds to >300 cells per condition. Means, SDs, maximum and minimum values are indicated. Significance was evaluated with unpaired t-test. n.s.>0.05. C) Assessment of FANCD2 knockdown efficiency by IF. Representative pictures of FANCD2 IF stainings (red) in HeLa cells transfected with the indicated siRNAs. D) IF analysis of ATRX (green) in G2 HeLa cells transfected with the indicated siRNAs. Two days post transfection cells were treated with DMSO or APH (0.2 μM) for 20h. E) HeLa cells were transfected with the indicated siRNAs and treated as in D). Box plots show the average of ATRX foci per G2 cell. Data shown corresponds to >300 cells per condition. Means, SDs, maximum and minimum values are indicated. Significance was evaluated with unpaired t-test. n.s.>0.05. *p≤0.05. F) Assessment of FANCD2 and ATRX single and double knockdown efficiency by IF. Representative pictures of FANCD2 (red) and ATRX (green) IF stainings in HeLa cells transfected with the indicated siRNAs. G) IF analysis of ATRX (green) and DAXX (red) in G2 cells. HeLa cells were treated with DMSO or APH (0.2μM) for 20h, and G2 cells were selected by DAPI content. Representative images are shown. H) Analysis of cell cycle profile of number of DAXX foci. HeLa cells were incubated with DMSO or APH (0.2 μM) for 20h and EdU (20 μM) was incorporated for the lasts 30min. DAPI and EdU profile were used to determine the different stages of cell cycle (G1, early, mid and late S, G2). Z-stack images were acquired to determine the number of foci per cell. The average number of DAXX foci per cell in each stage is illustrated in the box plots for DMSO (blue) and APH (green). Data shown corresponds to at least 600 cells per condition. Means, SDs, maximum and minimum values are indicated. Significance was evaluated with unpaired t-test. ****p≤0.0001. I) Assessment of ATRX and DAXX single and double knockdown efficiency by IF. Representative pictures of ATRX (green) and DAXX (red) IF stainings in HeLa cells transfected with the indicated siRNAs. Related to Figure 6.

Table S1. List of identified proteins with >1 peptide from the FANCD2 MS analysis.

Table S2. List of identified proteins, which are regulated 1.5-fold H/M in >50% of the experiments.

Table S3. List of identified proteins, which are regulated 1.5-fold H/L in at least 1 experiment.
